# Locating and Quantifying Methane Emissions by Inverse Analysis of Path-Integrated Concentration Data Using a Markov-Chain Monte Carlo Approach

**DOI:** 10.1021/acsearthspacechem.2c00093

**Published:** 2022-07-08

**Authors:** Damien Weidmann, Bill Hirst, Matthew Jones, Rutger Ijzermans, David Randell, Neil Macleod, Arun Kannath, Johnny Chu, Marcella Dean

**Affiliations:** † STFC Rutherford Appleton Laboratory, Harwell Campus, Didcot OX11 0QX, U.K.; ‡ MIRICO Ltd, Unit 6, Zephyr Building, Harwell Campus, Didcot OX11 0RL, U.K.; § Shell Global Solutions International B.V, Grasweg 31, Amsterdam 1031 HW, The Netherlands; ∥ Atmospheric Monitoring Sciences, Haringvlietstraat 27, Amsterdam 1078 JZ, The Netherlands

**Keywords:** methane emissions, emission spatial mapping, Markov-chain Monte Carlo, inverse methods, open path, laser dispersion spectroscopy, gas dispersion

## Abstract

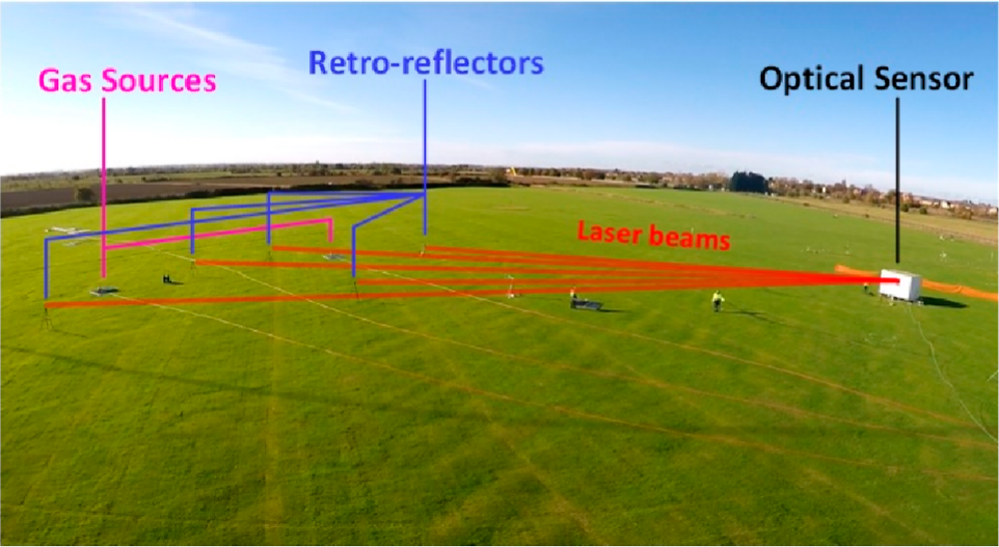

The action to reduce anthropogenic greenhouse gas emissions is severely constrained by the difficulty of locating sources and quantifying their emission rates. Methane emissions by the energy sector are of particular concern. We report results achieved with a new area monitoring approach using laser dispersion spectroscopy to measure path-averaged concentrations along multiple beams. The method is generally applicable to greenhouse gases, but this work is focused on methane. Nineteen calibrated methane releases in four distinct configurations, including three separate blind trials, were made within a flat test area of 175 m by 175 m. Using a Gaussian plume gas dispersion model, driven by wind velocity data, we calculate the data anticipated for hundreds of automatically proposed candidate source configurations. The Markov-chain Monte Carlo analysis finds source locations and emission rates whose calculated path-averaged concentrations are consistent with those measured and associated uncertainties. This approach found the correct number of sources and located them to be within <9 m in more than 75% of the cases. The relative accuracy of the mass emission rate results was highly correlated to the localization accuracy and better than 30% in 70% of the cases. The discrepancies for mass emission rates were <2 kg/h for 95% of the cases.

## Introduction

Methane and carbon dioxide are the dominant anthropogenic greenhouse gases responsible for warming the Earth’s atmosphere above its preindustrial era temperature.^1^ Their atmospheric concentrations are ∼1.88 parts per million by volume (ppmv) for methane,^2^ an increase of >1.0 ppmv in the past 250 years due to human activity,^3^ and ∼413 ppmv for CO_2_.^4^ The mass of CO_2_ in the atmosphere is 600 times that of methane, but methane is a dramatically stronger absorber of thermal radiation than CO_2_. Methane (with decay products) traps 58% of the heat trapped by CO_2_;^5^ thus, methane’s radiative forcing per kilogram (with decay products) is 350 times that of CO_2_. The Paris temperature targets of <2 °C temperature rise and preferably <1.5 °C are for this century, but methane’s atmospheric half-life is 9 years, while CO_2_’s is >100 years. Even if we could stop all emissions of CO_2_ from today (i.e., both natural and anthropogenic), most of the CO_2_ already in the atmosphere will still be there at the Paris Agreement deadline. In contrast, reducing global methane emissions will reduce radiative forcing within a decade, rather than a century: limiting temperature rise and the chance of triggering climate change “tipping points”. Global methane emissions have increased by 16% over the past decade;^6^ a rise consistent with a global warming of >4 °C by 2100. About 60% of methane emissions are anthropogenic; fossil fuels account for 35% of these; oil and gas production, distribution, and transport account for 23%.^7^

For the oil and gas sector, the most effective way to reduce methane emissions is leak detection and repair.^8^ Currently, this is done by labor-intensive leak detection of components using gas sensors; the actual emission rates are not measured. Most reported emissions are statistical estimates based on component counts, throughput, and simple average “estimates” of mass leakage rates per component (these are called inventory estimates). Many known-source categories are excluded, and inaccessible source locations, as well as unanticipated sources, are omitted.

Satellites with ground resolutions at kilometer scales have measured very large methane emissions of the order of ∼10 t/h.^9−11^ Those with ∼100 m ground resolution have attributed sources of ∼1 t/h.^12^ Airborne and drone surveys are typically episodic and do not provide information on intermittent emissions.^13,14^ These shortcomings have motivated the development of continuous monitoring solutions, for instance, by using a network of point sensors^15^ or long open-path integrated concentration measurements.^16^ Numerous sensing techniques and analysis methods were blind-tested as part of a methane measurement challenge for the oil and gas sector^17^ accuracies for mass emission rates were typically poor.^18^

We report a novel approach to methane emission mapping and quantification developed to provide long-term continuous area monitoring by a single fixed sensor. It combines the benefits of (1) open-path sensing with high-probability intersection with dispersing gases and low-variance high-precision measurements; (2) multipath sensing capturing spatial information; (3) use of mid-infrared (3.3 μm) laser dispersion spectroscopy providing a linear concentration response with immunity to weather and atmospheric scintillation; and (4) a Markov-chain Monte Carlo (MCMC) inference method estimating source locations and mass emission rates; sources outside the beam array are included. We report the performance of this approach for source localization and quantification using calibrated releases, including single-blind trials.

## Experimental Method

### Field Experiment

**Figure 1:**
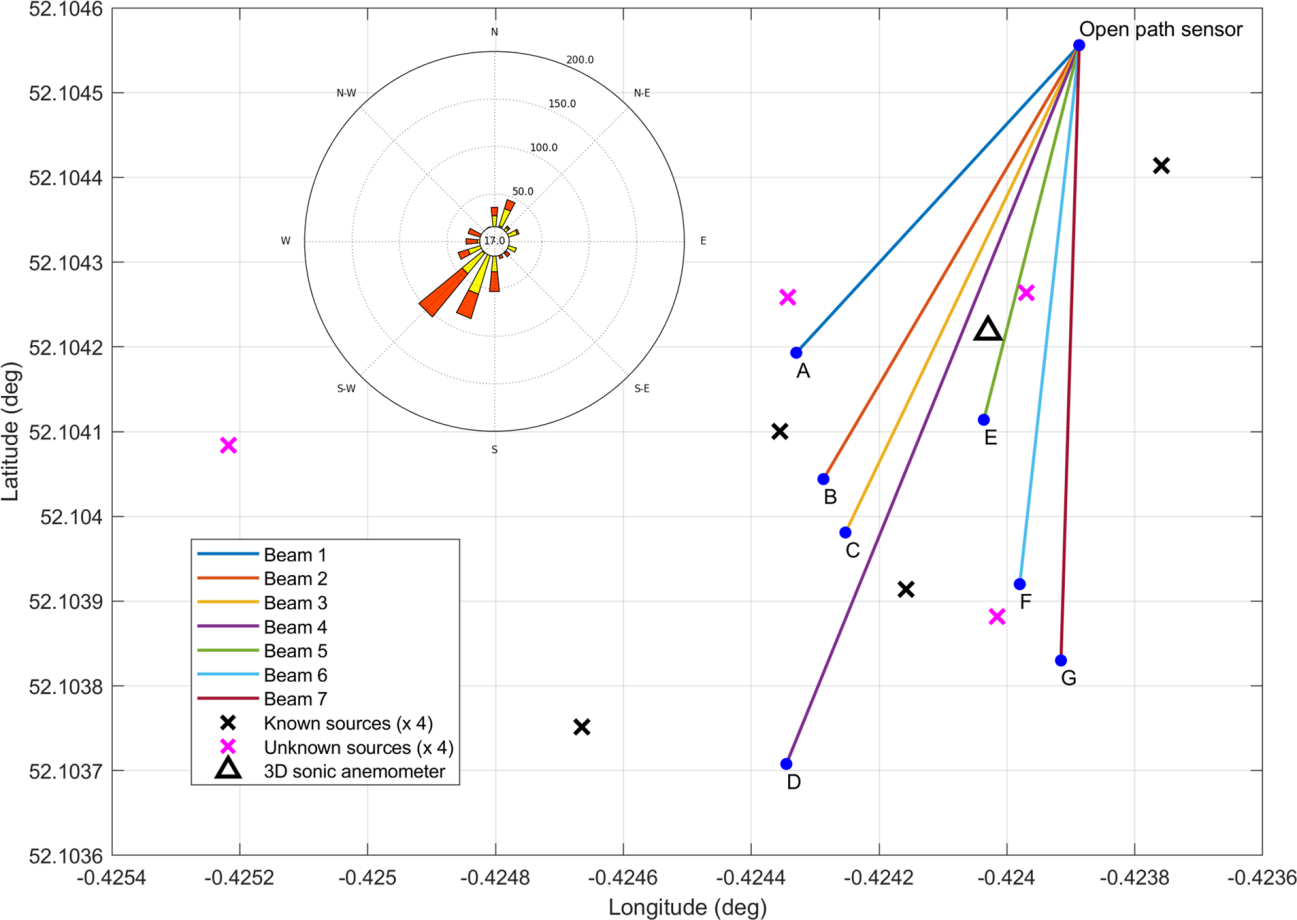
Experimental layout. Showing the full release area and locations of the open-path gas sensor, retroreflectors, and 3D sonic anemometer locations, as well as the known (black cross) and unknown (purple cross) source locations. Included in the top left corner is a wind rose displaying 2 years of daytime wind data. The longest beam is 99.4 m.

The calibrated methane gas release tests were done at the UK Met Office Meteorological Research Unit (MRU) at Cardington, Bedfordshire, UK, between 25th October and 1st November in 2017. This work followed earlier “proof-of-concept” tests that demonstrated the effectiveness of the technique.^19^ The new experiments incorporated improved sensor precision, refinements to the MCMC analysis, and inclusion of blind releases as a more objective measure of performance. The experimental arrangement is shown in Figure 1. The test area and surroundings were flat and unobstructed.

The orientation of the experimental beam array was based on 2 years of daytime wind data for the location; the prevailing wind is south-westerly (air moving from the southwest). The open-path sensor was installed in the northeast corner of the measurement area shown in Figure 1. Seven eye-safe beam paths radiate from the sensor to 5 cm aperture corner cube retroreflectors. From the demonstration study,^19^ seven beams were found to be a good compromise to target a few-meter spatial resolution on locating sources. Path lengths ranged from 99.43 to 50.26 m. A 3D ultrasonic anemometer was installed at the center of the beam array, and it measured wind velocity in the horizontal and vertical planes at 20 Hz. The beam height was 1.6 m above the ground; the anemometer was mounted at the same height and aligned to true north to ∼1° precision. Key experimental locations were mapped to ∼2 cm precision using a land surveying differential GPS. The sensor measures the path-averaged concentration (PAC) of each beam for 200 ms before steering to the next retroreflector; the scanning cycle for all seven beams was ∼3 s.

Our method measures time-averaged PAC on a timescale representative of the air transit time across the measurement area. The gas dispersion model provides an ensemble average of path-averaged concentrations on the same timescale. We use the Draxler Gaussian plume eddy dispersion model^20^ as all input parameters are directly measurable: unlike stability class-based models.

The gas releases were from perforated circular tubes (∼0.5 m inner diameter) covered by a 2 × 2 m^2^ frame of perforated sheet: so the gas escapes at the ground level with minimal momentum. The sources are connected by long hoses to the gas preparation unit, and this allows rapid and easy positioning anywhere within the test area. The gas was metered and tempered using calibrated mass flow controllers devised and operated by an independent team from the National Physical Laboratory (NPL), UK. The most remote source was 117 m from the sensor; gas release rates ranged from 3 to 5 kg/h per source, depending on the test. A typical test sequence comprised (a) 10 min of background CH_4_ measurements, (b) 1 h measurement of a stable release rate set of sources, and (c) another 10 min of background recovery measurement after ending the release. As the rate of change of wind direction is slow and unpredictable, we changed the release configurations to accumulate the maximum amount of wind variation in the limited experimental time available. This also dramatically reduced the amount of gas released. Consequently, each data set is concatenated from several tests across the few experimental days we had. The full list of releases made and their characteristics is provided in Table S1. No unexpected or unusually high safety hazards were encountered beyond the current practices of working with flammable gas. Conservative boundaries were set up to exclude personnel and potential ignition sources.

Three independent teams were involved. The instrument team was responsible for installing and configuring the gas sensor for the field measurements [MIRICO Ltd and Rutherford Appleton Laboratory (RAL)]. The gas release team was responsible for positioning the sources, providing the gas and calibrated flow control unit, and setting and maintaining stable gas release rates (NPL). The data analysis team was responsible for experimental design, the MCMC code, data analysis, and reporting results from the campaign [Shell and Atmospheric Monitoring Sciences (AMS)].

There were three main categories of tests with differing levels of information embargo maintained by NPL until after they received the analysis results from Shell and AMS: (1) four sources at known locations and a known emission strength of 5 kg/h (“known–known” scenario); (2) four sources at known locations but with unknown emission strengths (“known–unknown”); for this case, an additional blind scenario was included to gain insight on source location resolution performance, and this is referred to as “line and point”; and (3) unknown number of sources at unknown locations with unknown strengths (“unknown–unknown”). The latter scenario consists of a single blind test of the measurement method.

### Multibeam Open-Path Gas Sensor

The open-path gas sensor used for measuring PAC along multiple paths within the release area is based on the recent concept of high-resolution laser dispersion spectroscopy.^21^ In this approach, previously reported for CH_4_ measurements using both near-infrared^22^ and mid-infrared wavelengths,^23^ the “traditional” absorption spectrum is combined with high-resolution differential dispersion spectra of a selected CH_4_ molecular transition. This is used to obtain the path-integrated concentration. Differential dispersion adds the benefits of optical phase measurements: (i) greatly reduced sensitivity to light intensity fluctuations that occur for atmospheric measurements due to particulates, precipitation, scintillation, and opacity variations; (ii) true linearity to gas concentration; and (iii) enhanced dynamic range.^19^

In contrast to single-point measurement, long PAC includes inherent spatial averaging that smooths the stochastic-point concentration variance stemming from short-scale turbulence. It therefore provides a concentration estimate with uncertainties from eddy and advection variability reduced. Long path sensing also increases the chances to intersect the gas plume from an emission source. The multibeam configuration captures spatial information across the dispersion area. As the seven paths are measured sequentially in a shorter time than the gas transit time, variations of concentrations over the seven paths are captured. This information enables recovery of the likely source locations, which in turn constrains the associated emission rates of these sources. The beam fan deployed for this experiment was restricted to a full angle of 40°, instead of the preferred 360°, due to technical limitations of the scanning system used at the time. Likewise, the sensor optical system was configured for path lengths up to ∼100 m for the work while up to 400 m are now achievable.

### Inverse Solver Method: MCMC

The problem of recovering gas emission source locations and strengths, with given measurements of PAC and wind data, is ill-posed. Bayesian inference was used to generate solution estimates and uncertainties. Bayesian methods are particularly suited to inverse problems as they allow for the explicit inclusion of prior information about the structure of the solution: for example, we expect the source map to be sparse, the background concentration to evolve smoothly in time and to be correlated between beams, and so forth. The data analysis method and its theoretical basis have been thoroughly described in ref (19). The posterior distribution is derived from the data likelihood and the parameter prior distributions,^24^ and the posterior parameter space is explored through a MCMC algorithm. MCMC allows efficient exploration of the posterior distribution, without the need for an analytical expression for the posterior.^24^

In more detail, the vector ***y*** of measured concentration data points is assumed to be made up of the following components
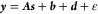
where ***s*** is the vector of source emission rates. ***A*** is a matrix of couplings, computed using the Gaussian plume model, mapping source emissions onto observed concentrations. ***b*** is the vector of background methane concentrations per observation. ***d*** is a vector of sensor calibration offset parameters. ϵ is a vector of measurement errors.

The above parameters are all unknown and must be estimated from the observed concentration data. To do this, we assume that the errors are Gaussian-distributed and make a set of appropriate prior assumptions for the unknown parameters. Given these assumptions, the posterior distribution is then constructed as follows
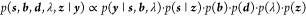
where the following additional parameters have also been introduced:

- λ is the set of parameters describing the precisions (inverse variances) of the measurement error terms ϵ (more detail given below).
- ***z*** is a vector of allocation parameters for the individual sources (described in more detail below).

More detail on the inversion algorithm and its mathematical background can be found in ref (19). For this first blind-trial evaluation, some refinements to the model and MCMC solver were made and compared with the initial demonstration^19^—these are described below:

- A more realistic prior distribution for the source emission rates was used, as compared with the initial demonstration. Rather than specifying an identical log-Gaussian prior for each possible source location and controlling the sparsity of the source map through the prior variance parameter, a so-called “slab and spike” Gaussian mixture prior is assumed,^25^ which can be a suitable model in the case of a few active hot spots distributed across an area. The slab and spike prior is a distribution made up of two Gaussian components: the “spike” describing the majority of the sources, which have an emission rate of close to 0, and the “slab” which allows a minority of sources to have a much wider range of possible emission rates. Each source from the spatial discretization (grid) must be allocated to one or other of these components. This allocation ***z*** is unknown and is estimated as part of the inversion process: a prior Bernoulli distribution is specified for the allocation independently per location, and the posterior allocation is sampled at each iteration of the MCMC chain from the resulting conditional distribution of the allocation (also a Bernoulli distribution, with probabilities obtained by combining the prior probabilities with the Gaussian probability density functions for the estimated emission rate at the current iteration). This prior is more realistic as a source emission map showing a few “hot spots” is expected (relative to the number of potential locations) when mapping emission sources. The characteristics of the estimated source map can now be controlled by specifying the prior variances of the slab and spike components and the prior percentage of sources which are believed to fall into each category.
- The measurement error prior distributions for the background and release periods were discriminated. During the background period, the measurement error is assumed to be due to the “true” measurement error, whereas that during gas releases is assumed to include errors due to measurement, real concentration inhomogeneities, and inadequacies of the dispersion model. Hence, more accurate identification of the background characteristics and beam offset parameters becomes possible and feeds into improving the quality of the inverse solution.
- The prior distribution for the source emission rates was a truncated Gaussian rather than a log-Gaussian, which enables a direct and efficient exploration of the posterior while maintaining the physical constraint that the emission rate must be positive. A truncated Gaussian prior was used for both the slab and spike components of the prior specification.

## Results

The results are reported for each test category of the experimental campaign, starting with the “known–known” case up to the “unknown–unknown” case. For each case, the key results of the inverse solution are presented in the form of “heat” maps showing mass emission rates and locations. The results are thoroughly reported for the first case as the format for the next two is similar.

### Known Source Locations–Known Emission Rates

The MCMC inverse solution is based on three data sets: releases 1, 7, and 13 (Table S1). To generate a solution, a number of inputs to the solver must be specified, most importantly the (slab and spike) prior standard deviations for the source emission rates, the prior allocation parameter for the slab and spike distribution, and the smoothness parameter for the evolution of the background concentration. Setting these inputs is nontrivial: they must be manually adapted in light of early results and the stability of solutions observed in the postrun graphical output. Initial inputs are chosen and adapted to obtain convergence and stability during the burn-in period of the MCMC sampling; the actual results are taken from the post-burn-in sample once stability is seen to have been established. The full list of input parameters for all cases reported is given in Table S2. 20,000 iterations of the MCMC chain are run in total, with 6000 of these being used as the post-burn-in sample for calculating posterior characteristics. Running these 20,000 iterations takes approximately 5 h on a desktop PC.

The adequacy of the Gaussian plume model is crucial to ensure a meaningful inversion, and the “known–known” case is a proxy way to evaluate this as part of the whole inversion approach and of the parameters setting. To that end, the temporal data series of methane PAC along the seven beams, as well as the wind vector data, were averaged over 30 s blocks to further reduce stochastic variation. The horizontal and vertical atmospheric turbulence intensities were calculated from the statistics of the 30 s averaged wind data. In addition, averaged data with a wind speed below 1.68 m/s were excluded as deemed to be of borderline validity for the Gaussian plume model; some users take 1.5 m/s as the effective limit. We originally chose 2 m/s as a conservative threshold but reduced this slightly to increase the volume of data and incorporate additional wind directions that would otherwise have been excluded.

The parameter settings of the slab and spike distribution prior determine the solution space explored. Typically, the prior for the number of “on” sources, κ, should be just a few percent of the spatial grid elements, and κ = 5% was chosen for this case. The standard deviation of the spike component, σ_spike_, should be close to zero as most of the map is expected not to emit; σ_spike_ = 0.076 kg/h was chosen. The standard deviation of the slab component, σ_slab_, is based on the expected mass emission rates occurring; σ_slab_ = 2.4 kg/h was chosen for an exploration of solutions up to ∼10 kg/h.

The emission rate prior parameters also interact to some degree with the inputs defining the anticipated background’s temporal smoothness. A weak source could be misinterpreted as background variation and vice versa. Again, finding the appropriate balance is iterative. After an initial choice of low κ and σ_spike_, the initial parameter describing the background flexibility parameter is tested using early MCMC burn-in runs (>10,000 iterations) to assess convergence and stability. It is considered valid if the background solution is smooth and slow as per the definition of the background. If not, another iteration after κ and σ_spike_ have been increased slightly is tested the same way. For the first data point, the background concentration prior distribution is a Gaussian centered at 2.1 ppmv, with a 100 ppbv standard deviation. The evolution of the background throughout each individual experiment is modeled as a Gaussian random walk, with an evolution standard deviation of 0.32 ppbv.

**Figure 2:**
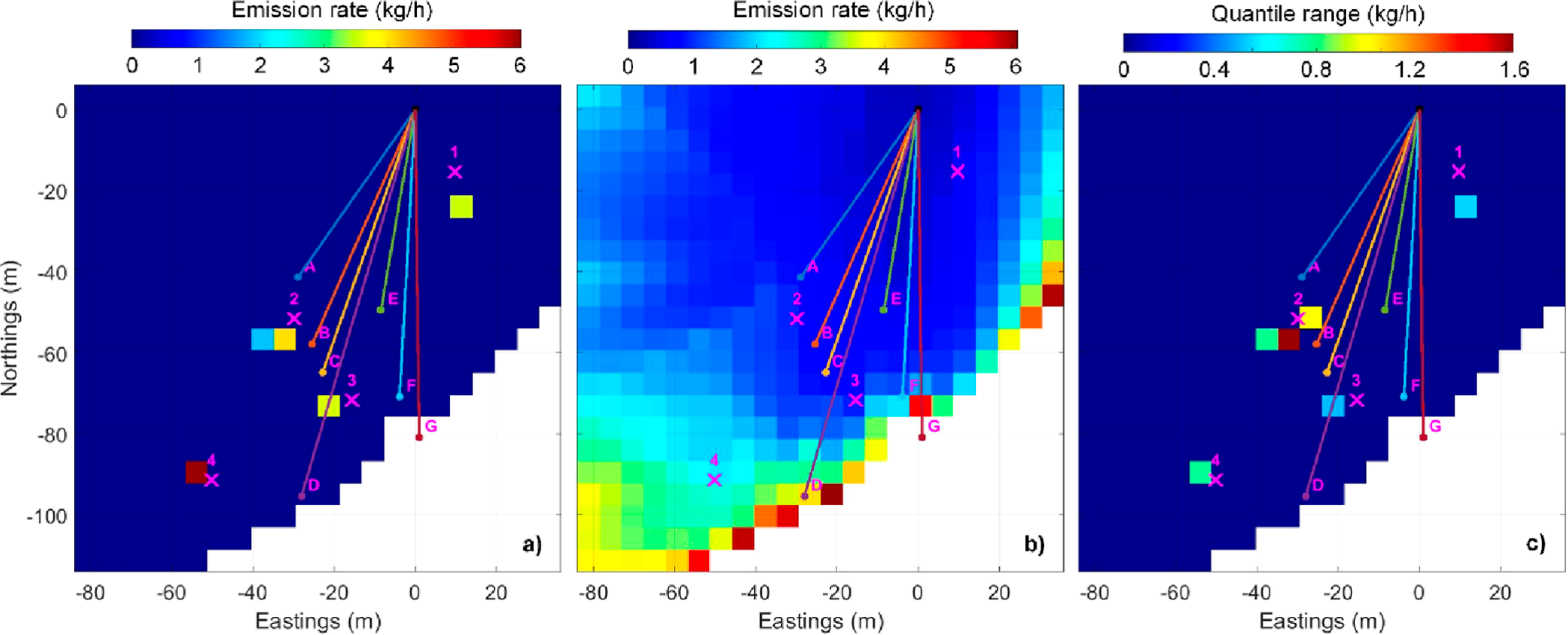
(a) “Heat” map of the median emission sources inferred by the MCMC solver. The optical beams of the gas sensor are shown as colored lines. The true source locations are each indicated by a purple “×”. (b) Coverage plot showing the minimum detectable source strength the sensor would be able to register at that location given the wind data. (c) Uncertainty map providing the 16–84% quantile range of the posterior distribution (±1 sigma in a normal distribution) of the source emission rates.

Using these input settings, the result of the MCMC analysis after 14,000 burn-in iterations is shown in Figure 2a with a map of the experiment and the open-path beams as colored lines. The known locations of sources are shown as purple crosses. Any source emitting less than 6 kg/h is considered undetectable by the measurement system if located in the white region (which is governed by wind conditions present in the data set used). Undetectable means in this context that considering the maximum coupling from the source to any of the beams, the concentration signal contribution is <10 ppb. Otherwise, a rainbow-color map indicates the median source emission rates per cell obtained from the inverse solution. The main results plot shows the spatial distribution of inferred sources, and their mass emission rates is further informed by Figure 2b showing the coverage sensitivity. Given the sensitivity of the whole measuring system (measurement plus inverse solution) and the meteorological conditions, the plot also shows the minimum source emission rate detectable for each cell of the area analyzed. This coverage is strongly dependent on the variety of wind directions occurring during the releases. Figure 2c shows the 16–84% quantile range (±1 sigma for a normal distribution) as an estimate of the source strength uncertainty. Further diagnostic plots help in assessing the quality of the solution, and these are shown in Figure S1.

The result has successfully resolved the four sources within the test area, and no “false” sources are present. Source 2 is inferred as more extended: being described by two grid cells in the solution. Table 1 quantitatively summarizes the results from the inverse solution. All results for source strength are <31% in absolute value from the actual and the localization is always better than two grid cells, and the grid cell resolution is 5.45 m.

**Table 1:** Summary of Localization and Quantification Results for the Case of Known Methane Emission Rates and Known Source Locations^a^

source	actual rate(kg/h)	inferred rate(kg/h)	inferred to actual relative difference (%)	distance actual to inferred (m)
1	5.11 ± 0.12	3.5 ± 0.5	–31	8.9
2	5.03 ± 0.04	5.9 ± 0.9	+17	6.6
3	5.06 ± 0.06	3.6 ± 0.3	–29	5.9
4	5.06 ± 0.03	6.1 ± 0.5	+20	4.1

a All uncertainties are 1 sigma.

The variety of wind conditions (direction, speed, and turbulence intensities) is critical in constraining the solution. The differing source location inference performance is related to the scarcity of favorable wind directions and velocities to constrain specific source locations, hence the importance of longer-term continuous data collection in capturing diverse wind conditions and improving the solution’s quality. For instance, the wind rose collected during the experiment and shown in Figure S2 confirms that few winds are available to constrain sources 1 and 2. To partially mitigate this, the wind-speed threshold, initially set to 2 m/s, was reduced to 1.68 m/s to include more data points with northerly winds at the risk of some potential compromise on Gaussian model validity.

Remotely mapping and quantifying source emission rates are challenging tasks, with relative uncertainties typically in the range of 100% or more (e.g., see the data in Ravikumar et al.^17^); these results demonstrate encouraging progress toward supporting targeted, efficient intervention to address persistent leaks and prioritize remedial action by the source emission rate. Determining the total emission rate from a facility is an additional goal for those seeking to reduce and report actual emissions. The MCMC method can also be used for this purpose. In this case, the distribution of the summed emission rates from all the cells can be calculated from the set of posterior samples generated by the MCMC chain. The median of the total emissions distribution will not be the sum of the median emission rates for the individual sources since the individual source emission rate distributions may be skewed and the emission rates of neighboring source locations are likely to be negatively correlated. For this test, the post-burn-in total emission posterior distribution is to be nearly Gaussian (Figure S3) with a mean of 19.3 kg/h and a standard deviation of 0.5 kg/h. The relative difference between the actual and the inferred total emission rate on the site is <4%, which is a very good result.

### Known Source Locations–Unknown Emission Rates

**Figure 3:**
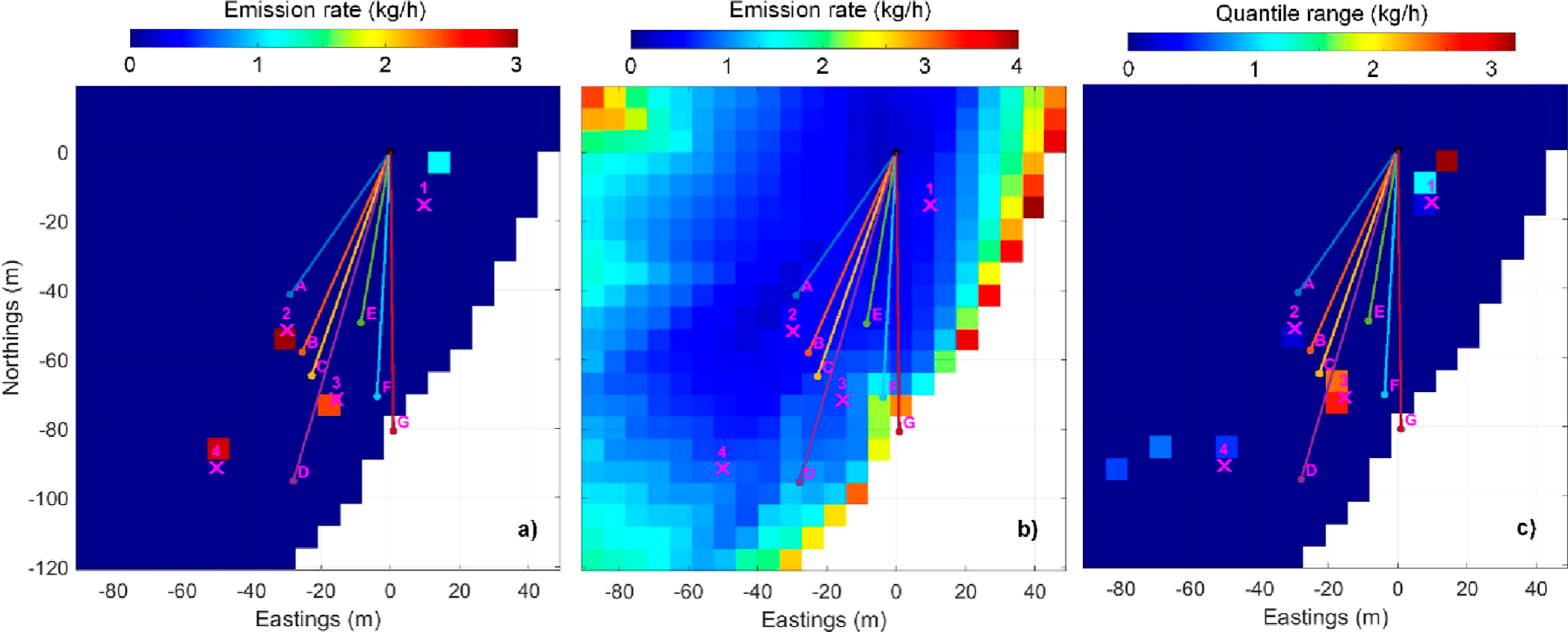
Similar MCMC inverse solution result maps to those shown in Figure 2 but for the case of known source locations with unknown mass emission rates.

The results from the second test category are presented the same way. The source locations were not changed from the previous case, but the team responsible for the gas releases (NPL) set the source mass flow controllers to values unknown to the other teams and not disclosed until these analysis results were reported to the NPL team. The experiment uses the data from releases 2, 8, 14, and 18 (cf. Table S1). The resulting maps from the MCMC inverse solution are shown in Figure 3. The quantitative summary is shown in the upper part of Table 2. Additional diagnostics from the MCMC inverse solution are provided in Figure S4.

**Table 2:** Summary of Results for Methane Source Localization and Quantification for the Case in Which Source Positions Were Known but Mass Emission Rates Were Unknown

source	actual rate (kg/h)	inferred rate (kg/h)	inferred to actual rate relative difference (%)	distance actual to inferred (m)
1	2.94 ± 0.10	1.2 ± 0.9	–59	12.8
2	2.98 ± 0.05	2.5 ± 0.8	–16	2.7
3	3.02 ± 0.05	3.1 ± 0.1	+3	2.6
4	3.01 ± 0.03	2.9 ± 0.4	–4	5.3

source	actual rate (kg/h)	inferred rate (kg/h)	inferred to actual rate relative difference (%)	distance actual to inferred (m)
1 (line)	4.4 ± 0.8	4.2 ± 0.3	–5	n/a
2	4.4 ± 0.8	3.5 ± 1.1	–20	3.2

For this second case, the quantification and localization results are very good, except for source 1. The larger uncertainties associated with source 1 are due to the extreme scarcity of the north-easterly winds necessary to constrain this location (i.e., to carry methane from source 1 across any of the beams). The estimated total mass emission rate is 13.5 kg/h with a sigma of 1.1 kg/h. Compared to the actual total mass emission rate of 11.95 kg/h, the inferred value is 13% higher than the actual rate.

**Figure 4:**
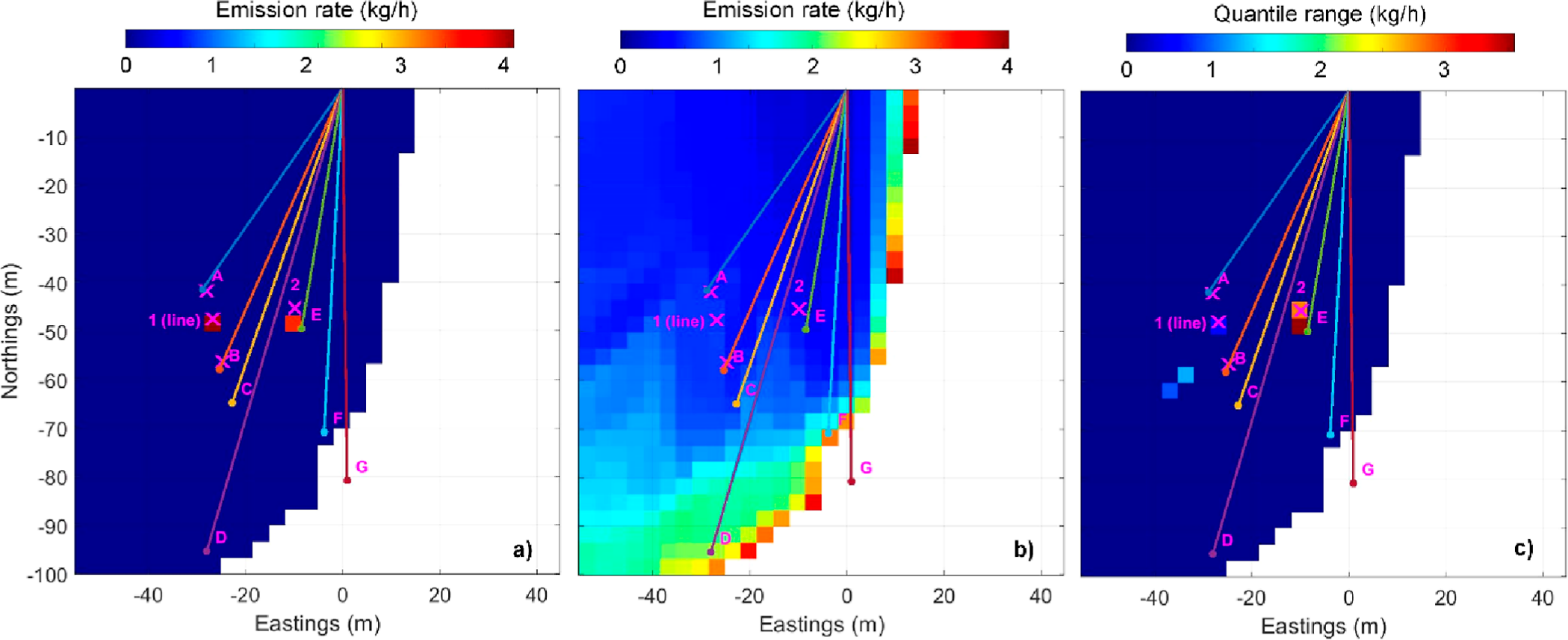
(a) MCMC inverse solution result maps for the separate case of known source locations and unknown mass emission rates, where the spatial resolution of the measurement technique was evaluated. Unknown number of sources and locations–unknown mass emission rates.

As part of the known–unknown experiment, a second geometry was devised to assess the spatial resolution of the emission mapping. Three sources were aligned between retroreflectors A and B, with a ∼5 m spacing. A fourth source was located 15 m northeast of that line. This case corresponds to the data from releases 5, 11, and 16 in Table S1. The resolution of the analysis mesh of cells was reduced to 3.3 m. The results are shown in Figure 4, and the quantitative summary is shown in the lower part of Table 2. The solution provided fairly good results for the location and good for total mass emissions but was unable to resolve the line of separate three sources. Instead, the solution attributed the line of three sources to a single source located at the line center. The most distant source 15 m away was resolved. The spatial resolution of the measurement system would seem to be between 5 m and 15 m. However, subsequent to reporting these results to the NPL gas handling team, they revealed at the time of disclosure that the source strengths had been changed unintentionally from 3 kg/h in the first release test to 5 kg/h in the two subsequent tests (cf. Table S1). The inconsistencies in emission rates within the data set will have increased uncertainties in the solution’s results shown in Table 2.

**Figure 5:**
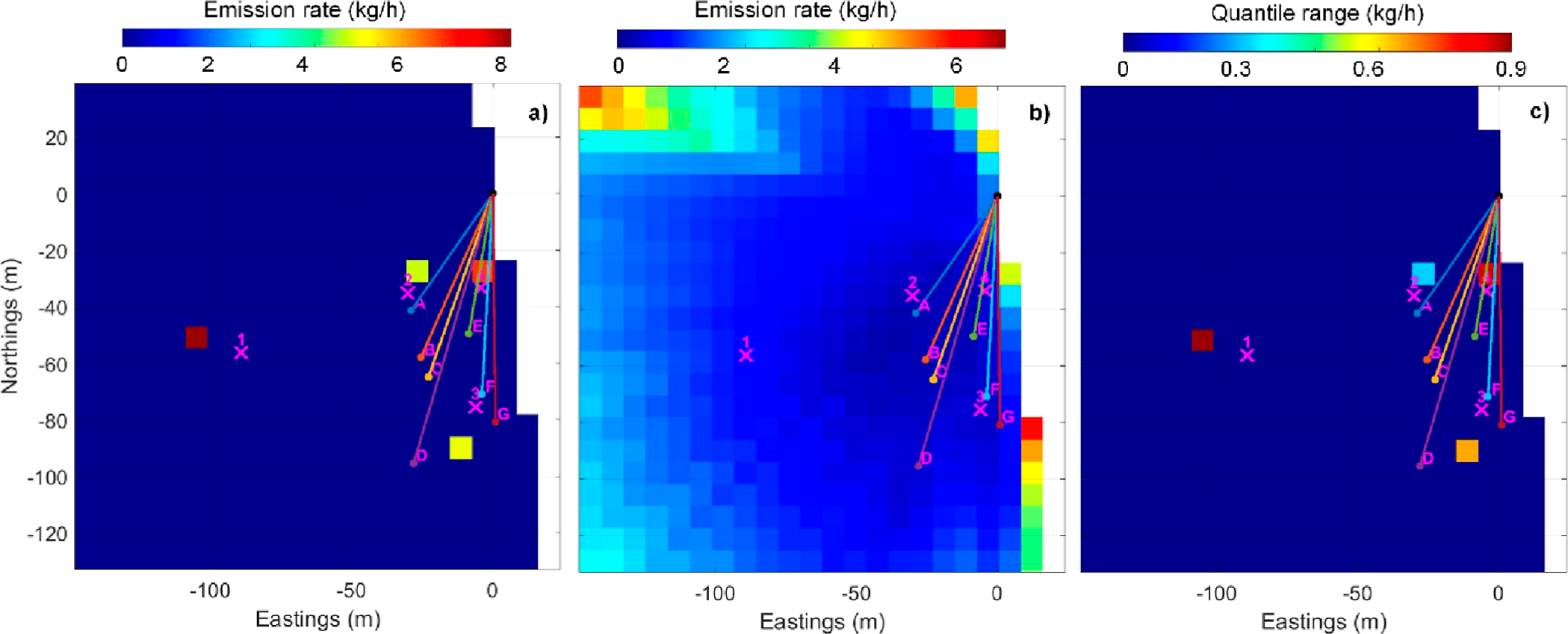
(a) Similar MCMC inversion result plots to those shown in Figure 2 but for the case of an unknown number of sources, of unknown mass emission rates, at unknown locations.

The last experiment corresponds to the fully blind case, which more closely resembles the method’s intended application of finding an unknown number of methane sources at unknown locations distributed within an area of interest. The data analysis team used the data from releases 3, 4, 9, 15, and 19 (Table S1). The full results of the blind MCMC inverse solution are given in Figure 5, with further diagnostic information in Figure S5. Table 3 provides a summary of the solution’s performance.

**Table 3:** Summary of Source Localization and Quantification Results for the Case of an Unknown Number of Sources, of Unknown Mass Emission Rates, at Unknown Locations

source	actual rate (kg/h)	inferred rate (kg/h)	inferred to actual rate relative difference	distance actual to inferred (m)
1	5.03 ± 0.04	8.7 ± 0.7	+73	16.7
2	5.03 ± 0.05	5.0 ± 0.3	–1	8.3
3	5.02 ± 0.07	5.2 ± 0.4	+4	15.3
4	5.04 ± 0.14	7.1 ± 0.5	+41	5.9

For this experiment, early solutions suggested that a distant Westerly source was involved and the scale of the inversion area had to be increased to include that distant source. To maintain the same number of grid cells, the size of the cells was increased to 7.8 m. The results for sources 2 and 3 are excellent. However, source 1 is given as about two grid cells further west than its true position. Source 1’s emission rate is overestimated by ∼70%; some of this will be due to compensating for the additional dispersion anticipated for that additional distance. The source 4 location is good, but its strength is overestimated by 40%. Source 4 is located at the edge of the coverage map and is likely to be insufficiently constrained by the wind data available.

In estimating the total emissions rate from the area, the MCMC estimation returns a bimodal distribution, suggesting competing solutions (Figure S5). The more probable solution, with the smaller uncertainty, appears to be 26.2 ± 0.5 kg/h. This is overestimated by 31% compared to the actual total emission rate revealed by the NPL team after result reporting.

## Conclusions

This study is the first single blind assessment of the new measurement technology combining multibeam open-path laser dispersion spectroscopy with MCMC inversion. The results are extremely promising in terms of total site emission estimation but also for the more demanding requirement of locating and quantifying individual methane sources at a facility level. Therefore, the method has the potential to play a significant role in the forthcoming international efforts in methane emission reduction, particularly targeting fugitive emissions from the oil and gas sector. The system presented was tested over short series of evaluation experiments. However, it aims to provide a unique tool for long-term real-time continuous monitoring of facilities, providing information on dynamics and temporal evolution. Besides, long-term continuous monitoring enhances the performance of the data inversion because of improved wind condition variability. The active sensor, due to its remote sensing capability, can be installed in the safe zone, while retroreflectors are fully passive components, configurable to optimize the spatial information to be retrieved. The spatial scale of the system is ideal to bridge the gap between satellite measurements and hand-held optical gas imaging cameras traditionally used for leak detection and repair campaigns on the ground. It is also foreseen that the technology will be critical in ground truthing and satellite data validation.

The results presented demonstrate encouraging progress toward addressing the fundamental barrier that has blocked methane emission reduction efforts to date: the difficulty of detecting, locating, and quantifying unknown sources’ mass emission rates. This progress is critical to switching from emission reporting based on inventory calculations to actual direct, trustworthy measurements. The need extends beyond the oil and gas sector and is highly relevant to other applications such as coal mining, landfills, agriculture, and biogas production. The availability of persistent emissions logging from facilities would help to reduce methane emissions effectively and efficiently. Work is being continued to progress our multibeam laser dispersion sensing approach on real facilities and evaluate its performance in more complex settings than in our reported work to date.

The next step is a long-term (3 months) continuous deployment at an actual mid-stream gas facility, with 24/7 operation. This deployment, which also envisages the inclusion of controlled release tests of short duration, will be used to further evaluate the potential of the hardware for long-term unattended operation, environmental robustness, and measurement reliability. It will also focus on evaluating the inversion methodology, particularly the limits of the Gaussian gas dispersion model in representing transport over a complex topography that includes buildings, piping, trees, and large equipment, none of which can be accounted for in such a simple model. Future development work will include evaluating alternative dispersion models better representing cluttered topography and their impact on the quality of gas emission source localization and quantification.

## Supporting Information Available

Additional experimental details on the gas releases, description of the MCMC inversion input parameters, and further diagnostics data and plots from the MCMC inversion (PDF)
