# Supplementary material for: Locating and Quantifying Methane Emissions by Inverse Analysis of Path-Integrated Concentration Data Using a Markov-Chain Monte Carlo Approach
Source: ACS Earth Space Chem. 2022 Jul 8;6(9):2190–8. doi: 10.1021/acsearthspacechem.2c00093 (PMC9483978; doi:10.1021/acsearthspacechem.2c00093)
Supplement: Supplementary file 1 — sp2c00093_si_001.pdf [file sp2c00093_si_001.pdf]

Supporting Information

Locating and quantifying methane emissions by inverse analysis of path-integrated concentration data using a Markov-chain Monte Carlo approach

Damien Weidmann[*, 1, 2], Bill Hirst[4], Matthew Jones[3], Rutger Ijzermans[3], David Randell[3], Neil Macleod[1], Arun Kannath[2], Johnny Chu[2], Marcella Dean[3]

1. STFC Rutherford Appleton Laboratory, Harwell Campus, Didcot, OX11 0QX, UK

2. MIRICO Ltd, Unit 6, Zephyr Building, Harwell Campus, Didcot, OX11 0RL, UK

3. Shell Global Solutions International B.V, Grasweg 31, 1031 HW, Amsterdam, The Netherlands

4. Atmospheric Monitoring Sciences, Haringvlietstraat 27, 1078 JZ, Amsterdam, The Netherlands

Summary

Number of pages: 11

Supplemental Figures: 5

Supplemental Tables: 2

* corresponding author: damien.weidmann@stfc.ac.uk

Table S1: List of gas releases and associated data. In the column 'Configuration', the abbreviations between brackets mean: (kk) known positions of all 4 releases, known mass release rate of 5 kg/hr per release; (ku) known positions of all 4 releases, unknown mass release rate of each release; (uu) unknown positions of releases, with unknown mass release rate. (line-point) indicates a sub-case of (k-u) aiming at testing the spatial resolution of the measurement method. The data in the last column that is marked with * was disclosed by the team from the National Physical Laboratory only after we completed all the data collection and analysis.

	**Date**	**Config.**	**Record start (UTC)**	**Gas start (UTC)**	**Gas stop (UTC)**	**Record stop (UTC)**	**Total emission rate (kg/h)**
1	25 Oct 2017	1 (kk)	9:22:00	9:43:00	10:51:00	11:05:00	4x5
7	27 Oct 2017	1 (kk)	9:57:00	10:14:00	11:14:00	11:25:00	4x5
12	30 Oct 2017	1 (kk)	13:32:00	13:52:00	14:25:00	14:43:00	4x5
13	31 Oct 2017	1 (kk)	10:20:00	10:38:00	11:41:00	11:55:00	4x5
17	1 Nov 2017	1 (kk)	8:05:00	8:20:00	9:36:00	9:51:00	4x5
2	25 Oct 2017	2 (ku)	11:10:00	11:29:00	12:36:00	12:50:00	4x3*
8	27 Oct 2017	2 (ku)	11:29:00	11:47:00	12:47:00	1:02:00	4x3*
14	31 Oct 2017	2 (ku)	12:10:00	12:28:00	13:32:00	13:48:00	4x3*
18	1 Nov 2017	2 (ku)	10:05:00	10:20:00	11:20:00	11:35:00	4x3*
3	25 Oct 2017	3 (uu)	15:05:00	15:14:00	16:36:00	16:47:00	4x5*
4	26 Oct 2017	3 (uu)	10:17:00	10:40:00	11:43:00	11:55:00	4x5*
9	27 Oct 2017	3 (uu)	14:22:00	14:23:00	14:46:00	15:00:00	4x5*
15	31 Oct 2017	3 (uu)	14:04:00	14:22:00	15:25:00	15:36:00	4x5*
19	1 Nov 2017	3 (uu)	11:40:00	11:53:00	12:53:00	13:08:00	4x5*
5	26 Oct 2017	2 (line-point)	13:20:00	13:37:00	14:56:00	15:07:00	2x3*
11	30 Oct 2017	2 (line-point)	10:58:00	11:19:00	12:26:00	12:40:00	2x5*
16	31 Oct 2017	2 (line-point)	15:47:00	16:00:00	17:00:00	17:15:00	2x5*
6	27 Oct 2017	outside program	9:11:00	9:31:00	9:39:00	9:52:00	-
10	30 Oct 2017	Calibration	10:17:00	10:20:00	10:52:00	10:58:00	-

Table S2: Details of input parameter settings used in the MCMC inversion for each case considered during the field experiment.

Parameter (unit)	k-k	k-u	k-u (line)	u-u	Comments
Area considered (m^2)	120 x 120	140 x 140	100 x 100	172 x 172	Size of the domain, should be large enough to encompass all sources.
Number of grid cells	22 x 22	22 x 22	30 x 30	22 x 22	The number of grid cells indicates the resolution on source locations, but the number has to be chosen judiciously in relation to the amount of data points available: the mass flow each grid point represents a variable in the MCMC algorithm.
Grid cell size (m^2)	5.45 x 5.45	6.37 x 6.37	3.33 x 3.33	7.82 x 7.82	Area considered divided by the number of grid cells.
Averaging time (s)	30	30	30	60	The averaging time should be similar to the gas transit time over the beams. This eliminates the high-frequency fluctuations in the methane concentration and wind measurements due to turbulence in the atmosphere. For the smoothed data, the Gaussian plume model used in the MCMC is appropriate.
Wind speed threshold (m/s)	1.68	1.5	1.5	1.5	Trade-off between Gaussian plume model accuracy and amount of wind diversity constraining inversion.
Standard deviation of the slab distribution (kg/hr)	2.405	2.405	2.405	5.377	governs range of source emission rates considered likely given allocation to the slab distribution (high emission rate sources)

Standard deviation of the spike distribution (kg/hr)	0.076	0.076	0.076	0.076	governs range of source emission rates considered likely given allocation to the spike distribution (low/zero emission rate sources)
Prior for the percentage of sources switched on (%)	5	25	25	1	The prior gives a first estimate of the percentage of grid cells that have non-zero mass emission rate. In general, the results from the MCMC are not dependent on the initial prior, but the parameter can be changed to reduce the number of MCMC iterations required to reach a converged solution.
Background prior mean (ppm)	2.1	2.1	2.1	2.1	
Background prior precision (ppb)	316	447	316	316	Standard deviation of the evolution of a Gaussian random walk
Background standard deviation at time 0 (ppb)	100	100	100	100	
Maximum emission rate (kg/hr)	6	4	4	7	
Burn-in iterations	14,000	8,000	10,000	18,000	Number of iterations needed to obtain a stable solution. These iterations allow the MCMC algorithm to explore the solution space.
Iterations for statistics collection	6,000	2,000	15,000	4,000	Number of iterations used to gather statistics for reporting.

Figure S1: Case of known source locations and known source emission rates (k-k). Left: Probability densities of the measurement precision of the optical analyzer for all beams. The probability densities appear near Gaussian, as expected from a realistic solution, with a consistent one sigma of ~7 ppb. Right: Standard deviation of the path averaged concentration residuals (i.e. the discrepancy between the measured path averaged concentration and modelled one in MCMC solution) during the run. Beam one has been excluded as it does not receive any methane signal from the sources. Again, distributions are nearly Gaussian with a one sigma of ~50 ppb.

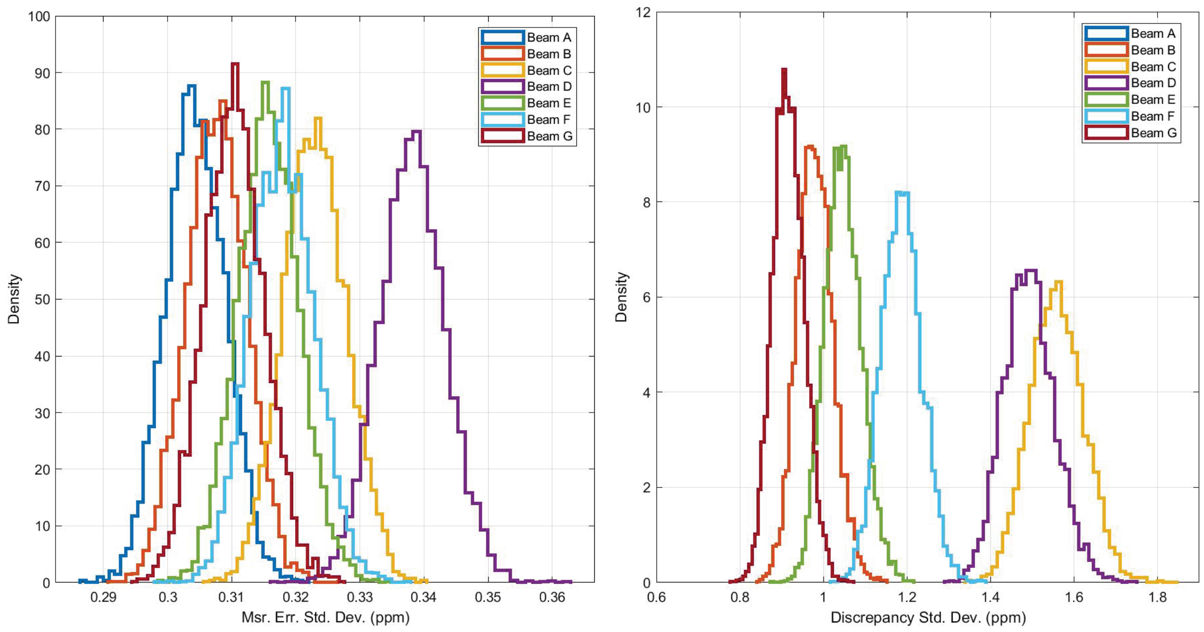

Figure S2::Case of known source locations and known source emission rates (k-k). Wind rose during releases 1, 7 and 13, showing the 30 s-averaged data points as a function of wind direction and windspeed.

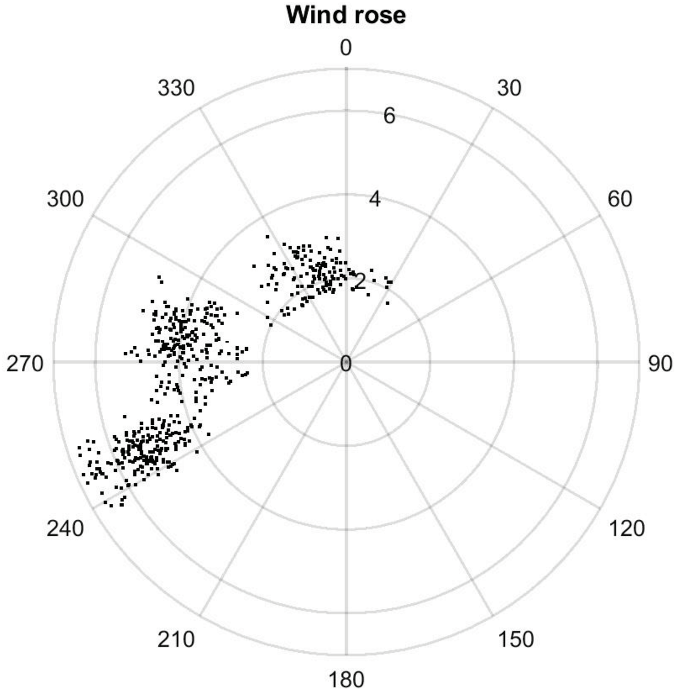

Figure S3: Case of known source locations and known source emission rates (k-k). Left: development of the total emission rate during the MCMC run. The first 14,000 iterations were used to obtain a stable solution (burn-in, grayed in the plot); only the results from the last 6,000 iterations were used to gather statistics. Right: probability density of the total emissions rate estimated.

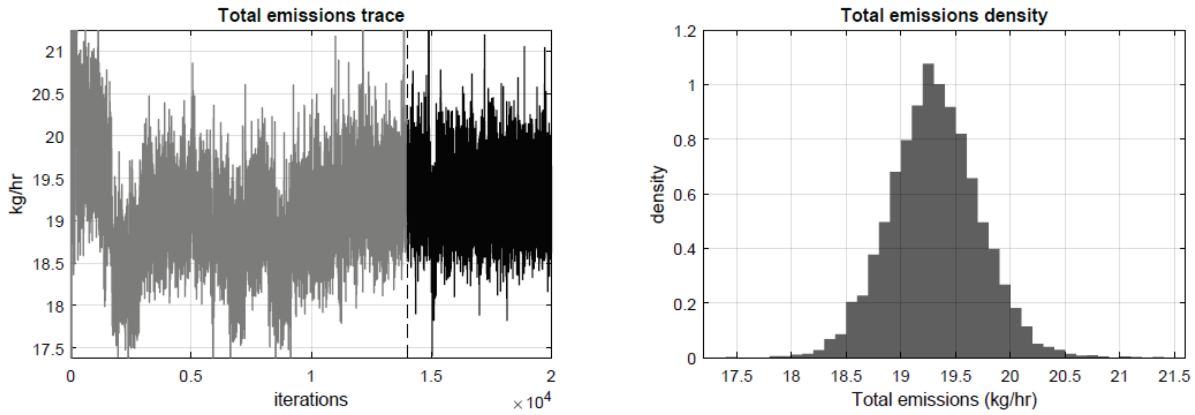

Figure S4: Additional diagnostic data for the case of known source locations and unknown source emission rates (k-u). Top left: Probability densities of the measurement precision of the optical analyzer for all beams. Top right: Standard deviation of the path averaged concentration residuals. Middle left, total emission rate sampling during the MCMC run. Middle right, probability density of the total emissions rate estimated. Bottom left, Wind rose showing the 30 s-averaged data points as a function of wind direction and windspeed.

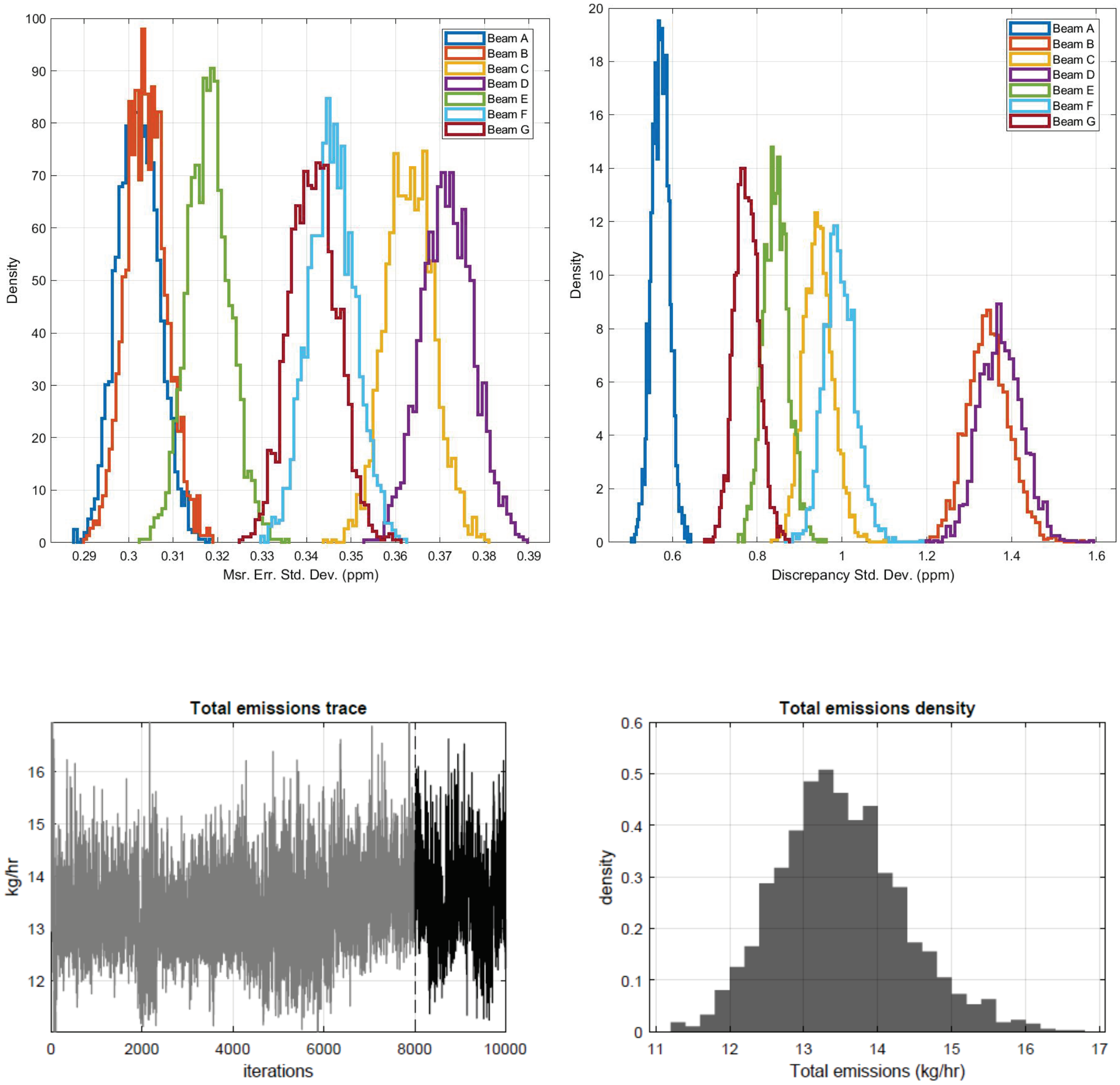

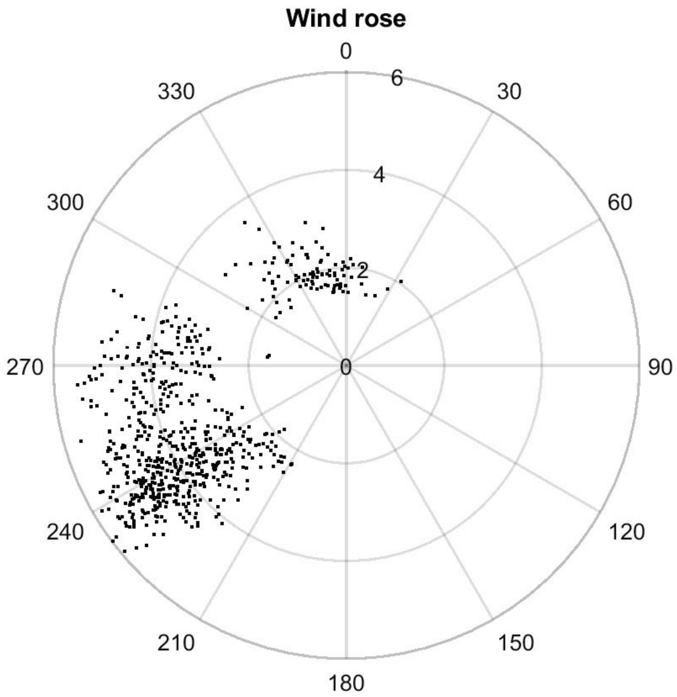

Wind rose
0
30
60
90
120
150
180
210
240
270
300
330
0
2
4
6

Figure S5: Additional diagnostic data for the case of known source locations and unknown source emission rates (u-u). Top left: Probability densities of the measurement precision of the optical analyzer for all beams. Top right: Standard deviation of the path averaged concentration residuals. Middle left, total emission rate sampling during the MCMC run. Middle right, probability density of the total emissions rate estimated. Bottom left, Wind rose showing the 30 s-averaged data points as a function of wind direction and windspeed.

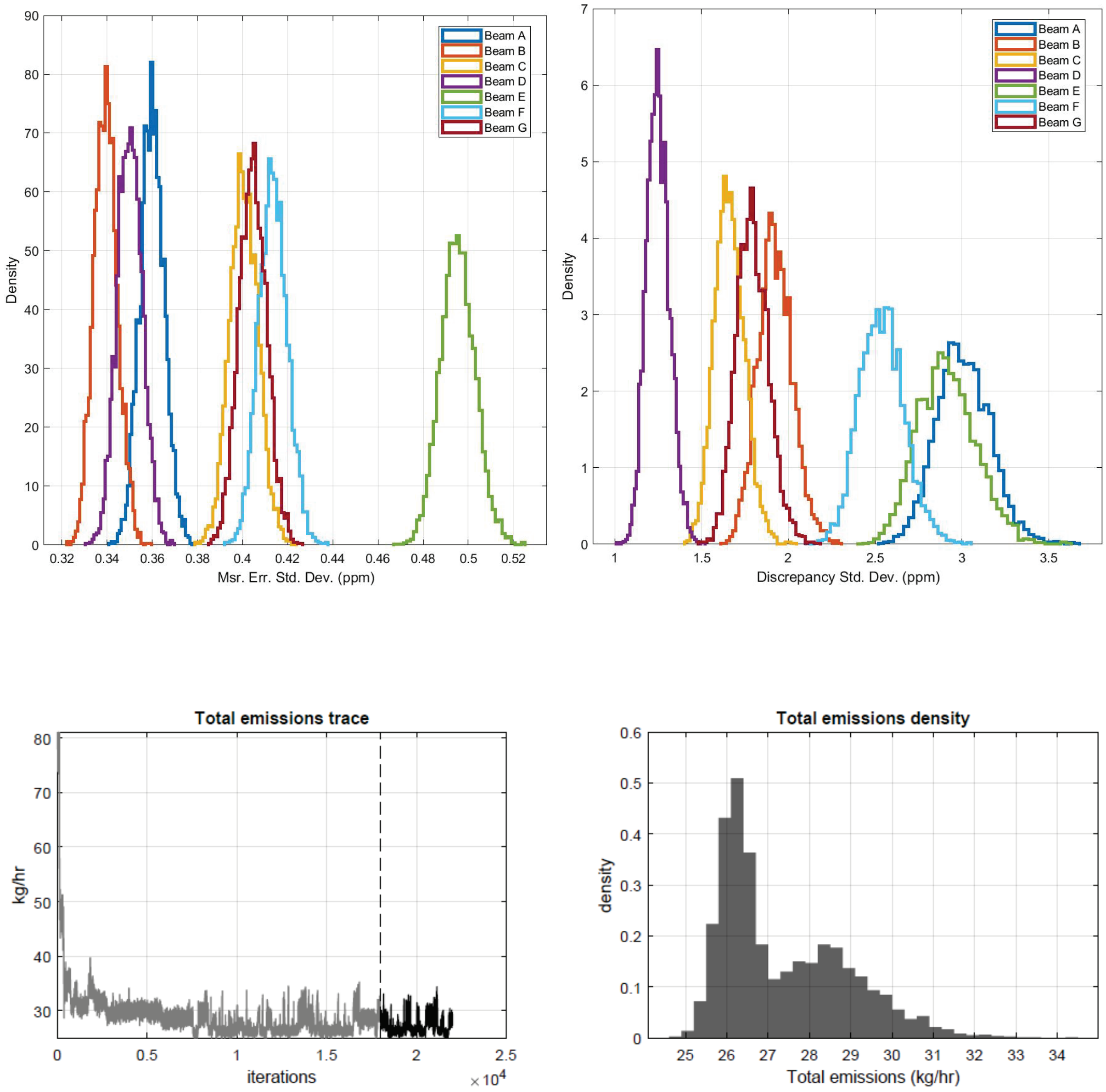

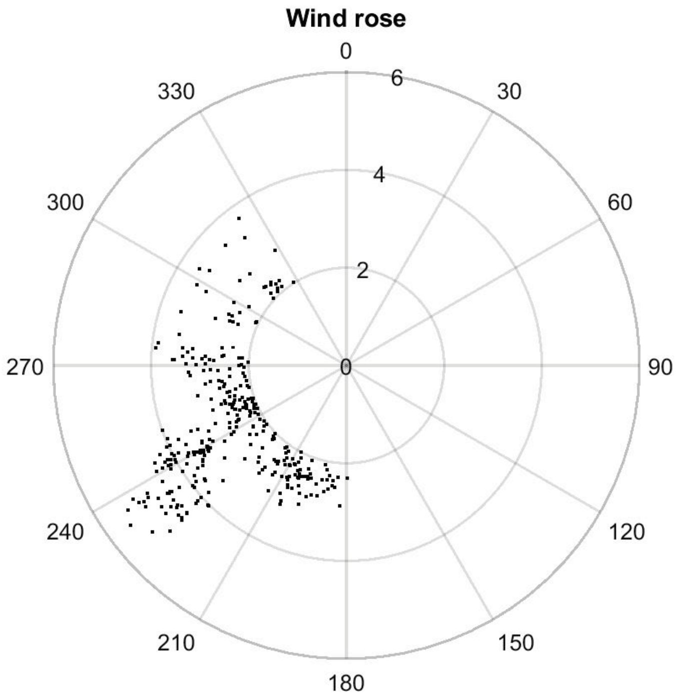
Wind rose
0
6
30
60
90
120
150
180
210
240
270
300
330
4
2
0
